# Effects of Starvation on the Levels of Triglycerides, Diacylglycerol, and Activity of Lipase in Male and Female Drosophila Melanogaster

**DOI:** 10.1155/2021/5583114

**Published:** 2021-03-25

**Authors:** Ved Chauhan, Asaba Anis, Abha Chauhan

**Affiliations:** New York State Institute for Basic Research in Developmental Disabilities, Staten Island, New York, USA

## Abstract

We studied the effects of starvation on changes in neutral lipids in male and female *Drosophila melanogaster* (fruit fly) at different ages. When flies were subjected to starvation, the mortality rate was observed to be age- and gender-dependent: male flies died earlier as compared to female flies, and older flies died earlier than younger flies. There was an increase in the number of dead flies and the levels of diacylglycerol (DG) with starvation time. This increase in DG was observed much earlier in male flies as compared to female flies, which correlated with earlier death in male flies during starvation in comparison to female flies. We also analyzed the levels of triglycerides (TG) and lipase activity during starvation of flies. The levels of TG decreased depending upon the duration of starvation in both male and female flies. Interestingly, we observed that like DG, there was also an increase in lipase activity due to starvation, which also correlated with earlier death in male flies as compared to female flies. Our results suggest that increase in DG levels and lipase activity due to starvation may be the main cause of death in the flies.

## 1. Introduction

Starvation is the most severe form of malnutrition. In starvation, severe deficiency in energy intake induces a metabolic response focused on the subsistence of the vital organs to allow for survival of the affected individual. It is estimated that about 805 million people suffer from malnutrition. Approximately 45% of death in children under 5 years of age can be correlated with starvation [1, 2].

It is common among human to go through dietary restriction, fasting, and starvation. Normally, the extension of lifespan and promotion of healthy brain aging are through dietary restriction. Restriction of food has been shown to extend the lifespan of multiple organisms including *C. elegans*, mice, and nonhuman primates [3–6]. One of the two modes of dietary restriction is caloric restriction (reduction in calories without malnutrition), and the second method is through intermittent fasting, where a normal diet is eaten but there are periods without access to food. The starvation could be for a short period (which may affect health) or for a long period, leading to the death of the individual.

*Drosophila melanogaster* (fruit fly) seems to be a good model for starvation studies. Nearly 75% of disease-related genes in humans have functional orthologs in fruit fly [7]. *Drosophila* and humans share similarities in overall brain architecture and show strong conservation in the mechanisms of neuronal development and signaling, as well as the main neurotransmitter systems [8, 9]. Furthermore, *Drosophila* exhibits a wide range of complex and sophisticated behaviors, such as circadian rhythms, sleep, learning and memory, courtship, feeding, and aggression that are shared by mammals [8, 9]. However, *Drosophila* has a short lifespan and high fecundity. The significant levels of conservation between *Drosophila* and humans make it an outstanding model to study longevity and physiological processes associated with aging [10, 11]. As most organisms age, several changes occur that may include reduced responses to stress, altered metabolic profiles, and alterations in sleep behaviors.

There are many factors that participate in metabolic processes during starvation or malnutrition. One such factor is the target of rapamycin (TOR) signaling pathway that gets triggered by nutritional factors. TOR pathway is also related to a link between nutrition and longevity [12]. It is reported that TOR inhibition in stem cells and progenitor cells shortens the lifespan of Drosophila on regular diet as well as under malnutrition conditions [13]. In addition, Drosophila appetite is influenced by insulin-like peptide (dilp) genes that also regulate the lifespan in Drosophila. There are eight dilp known that are involved in the accumulation of storage metabolites such as triglycerides (TG), glycogen, glucose and trehalose in fruit fly bodies, and carbohydrates in hemolymph. While dilp2 mutation resulted in decreased glycogen levels, lack of dilp3 led to a significant increase in circulating trehalose and glycogen levels, particularly at low protein consumption. On the other hand, lack of dilp5 caused decreased levels of glycogen and TG on all diets, and knockout of dilp7 resulted in increased glycogen levels and decreased TG levels at low protein consumption [14]. Yuan et al. [15] reported that 20-hydroxyecdysone (20E) antagonized insulin/insulin-like growth factor signaling through the AMP-activated protein kinase (AMPK)- protein phosphatase 2A (PP2A) axis in the fat body and suppressed the growth rate in Drosophila.

There is also a relationship between hunger and synaptic signaling from olfactory sensory neurons (OSNs) to brain neurons. Lushchak et al. [16] reported that increasing the OSN signaling by genetic manipulation elevates carbohydrates and diminishes lipids. It has been reported that glucose and fructose affect carbohydrate and lipid metabolism differently by modulation of insulin/insulin-like growth factor signaling [17].

Lipids (energy source) in animals are regulated by lipogenesis and lipolysis in response to food availability. In *Drosophila*, lipid reserves are mainly stored as TG in the fat body. The lipolytic factors are conserved between insects and mammals [18]. TG is the main source of energy during starvation. In this study, we investigated how starvation affects the levels of TG and diacylglycerol (DG) and activity of lipase during aging in both male and female flies. We selected 2-day-old flies (2 d), 12-day-old flies (12 d), and 23-day-old flies (23 d) for these studies and observed that starvation has different impact depending upon the age and gender of the flies. During starvation, male flies of the same age group died earlier as compared to female flies. The effect of starvation on mortality rate in both male and female flies was also age-dependent: 23 d flies > 12 d flies > 2 d flies.

## 2. Materials and Methods

### 2.1. Drosophila melanogaster

Wild-type Oregon-R *Drosophila melanogaster* stocks were maintained at 25°C on a standard cornmeal diet (Jazz-mix *Drosophila* food, Fisher Scientific, Pittsburgh, PA, USA) under 12 h : 12 h light and dark cycle.

#### 2.1.1. Starvation Procedure

Male and female flies were separated, and 10 flies were kept in each glass tube (16 × 100 mm) with a cotton plug (total of six tubes, i.e., one control tube and five tubes for varying starvation time period). The tubes were kept for starvation in the incubator (temperature, 25°C; humidity 65%) with 12 h : 12 h light and dark cycle. The tubes were removed from the incubator at various time intervals as indicated in the figure legends, and the number of dead flies was counted. The tubes containing dead and alive flies at each time period of starvation were stored at -20°C and later used for the estimation of neutral lipids and lipase activity. The final phase of starvation was considered when the maximum number of flies had died, i.e., 33 h for male and 48-57 h for female flies. All male flies were dead at 48 h, but we continued incubation until 57 h to study whether alterations in the levels of neural lipids and activity of lipase continued after death.

#### 2.1.2. Extraction of Lipids

The flies in each tube, i.e., 10 flies, were washed with distilled water and wiped with a paper towel to remove the water. All the flies were homogenized in 300 *μ*l of chloroform : methanol (2 : 1, *V*/*V*) and then mixed with 60 *μ*l of distilled water. The tubes were centrifuged at 10,000 rpm for 10 min. The upper water phase along with the proteins at the interphase of water was removed, and the organic phase sample was used for the separation of lipids by thin layer chromatography.

#### 2.1.3. Thin Layer Chromatography of Lipids

For separating neutral lipids, i.e., TG and DG, 25 *μ*l of organic phase was loaded on Silica gel 60 plates, and lipids were separated by running the plate in solvent mixture containing petroleum ether : solvent ether : acetic acid (90 : 10 : 1, *V*/*V*) as described earlier [19]. Different lipid spots were visualized by keeping the plate in the iodine chamber, followed by scanning. The densities of spots were measured by using Multi Gauge V3.0 software (Fujifilm).

#### 2.1.4. Lipase Activity

It was measured by Abcam kit (Cat # ab102524). 10 flies (starved), and 10 control flies (no starvation) were washed with distilled water and wiped with a paper towel to remove the water. The flies were then homogenized in 100 *μ*l assay buffer (provided in Abcam kit), followed by centrifugation at 10,000 rpm for 10 min. The supernatants were collected and transferred to clean tubes and kept on ice. 10 *μ*l of sample was used for assaying lipase activity. Lipase activity was calculated as nmol of glycerol per mg protein.

#### 2.1.5. Protein Measurement

The protein estimation was done using Quick Start Bradford Protein Assay kit (BioRad) following its protocol. In brief, protein standard (bovine serum albumin) and experimental samples were added to the wells in a microplate and mixed with a dye reagent. After 10 min of incubation at room temperature, optical density was measured using SpectraMax M5 (Molecular Devices).

#### 2.1.6. Statistical Analysis

The data in the starvation group vs. that in control group were analyzed by two-way ANOVA and unpaired Student's *t*-test.

## 3. Results

### 3.1. Effect of Starvation as a Function of Aging in Male and Female Flies

**Figure 1:**
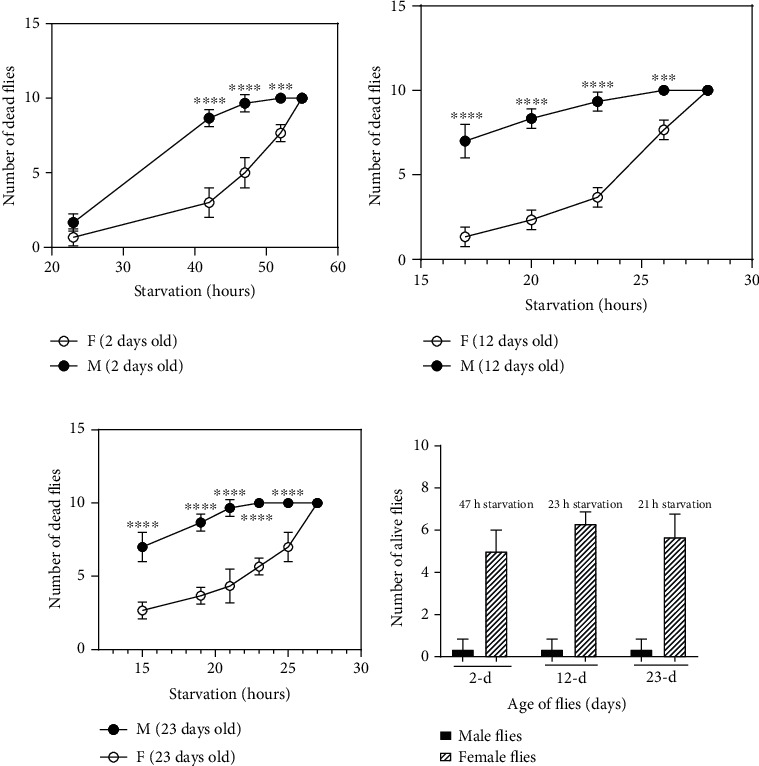
Starvation affects the survival of flies based on age and gender. Male (M) and female (F) flies of different ages (2 d, 12 d, and 23 d) were kept under starvation. The survival of flies during starvation was age-dependent: 2 d flies (a) survived longer than 12 d (b) and 23 d (c) male and female flies. Similarly, 12 d flies (b) survived longer than 23 d (c) male and female flies. During starvation, male flies at all ages died faster than female flies of the same age group. (d) The number of flies that had survived (out of 10 male and 10 female flies) at a specific starvation time period shown. The data is presented as the mean ± SD. It was analyzed by two-way ANOVA with Sidak's multiple comparison test between male and female flies. ∗∗∗∗ denotes *p* < 0.0001, and ∗∗∗ denotes *p* < 0.001.

We selected 2-day-old flies (2 d, Figure 1(a)), 12-day-old flies (12 d, Figure 1(b)), and 23-day-old flies (23 d, Figure 1(c)) to study the effects of starvation. Upon starvation, all male flies were dead at 52 h in 2 d flies, at 26 h in 12 d flies, and at 23 h in 23 d flies, while in female flies, all flies were observed to be dead at 55 h in 2 d flies, at 28 h in 12 d flies, and 27 h in 23 d flies. This data suggests that 2 d flies survived longer (Figure 1(a)) than 12 d flies (Figure 1(b)) and 23 d flies (Figure 1(c)). There was a significant difference of starvation in male flies as compared to female flies in 2 d, 12 d, and 23 d old flies (*p* < 0.0001). It was observed that female flies survived longer than male flies in all age groups studied.  Figure 1(d) shows the number of starved female flies that had survived at a starvation time when all the corresponding male flies were observed to be dead in different age groups. It was observed that 50-60% of female flies were still alive when 90-100% of male flies had died due to starvation in all age groups.

### 3.2. Levels of TG and DG in 2 d Old Male and Female Flies during Starvation

**Figure 2:**
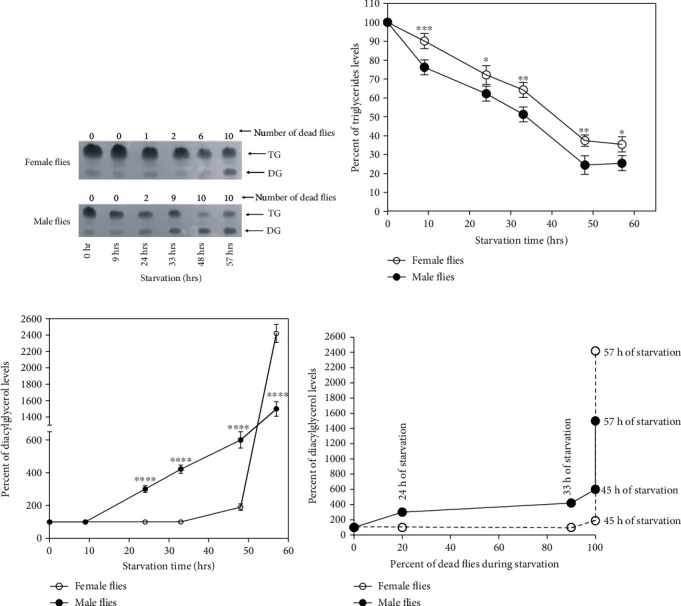
Levels of TG and DG in 2 d old male and female flies during starvation. Lipids were separated on thin layer chromatography at different time periods after the male and female flies were subjected to starvation (a). The density of the TG spots (b) and DG spots (c) (percent of control) as a function of starvation time was measured. Levels of TG in both male and female flies decreased depending upon the duration of starvation. There was a significant increase in levels of DG in final phase of starvation (48-57 h) in female flies (c). In male flies, DG levels continued to increase after death from 48 to 57 h (all 10 male flies were dead at 48 h of starvation) (c). The percent of DG levels vs. percent of dead male and female flies at different starvation period is shown in (d). The data is representative of three experiments and is presented as the mean ± SD. It was analyzed by two-way ANOVA with Sidak's multiple comparison test. ∗∗∗∗ denotes *p* < 0.0001, ∗∗∗ denotes *p* < 0.001, ∗∗ denotes *p* < 0.01, and ∗ denotes *p* < 0.05.

The levels of TG decreased and that of DG increased depending on the duration of the starvation time in 2 d male and female flies (Figure 2(a)). The decrease in TG levels was more in male flies compared to female flies upon starvation (Figure 2(b)). There was a significant difference between male and female flies at all time periods of starvation studied. At 57 h of starvation, levels of TG in male flies were observed to be only 22% of control flies. In female flies, about 35% of TG was still present at 57 h of starvation. DG appeared much earlier in male flies as compared to female flies corresponding to earlier death in male flies during starvation (Figure 2(c)). In male flies, DG levels increased by 3x at 24 h and continued to increase in a linear manner and were 15x at 57 h (final hour of starvation). In female flies, a significant increase in DG levels by 24x was observed during final hours of starvation from 48 to 57 h. In both male and female flies, the increase in DG levels correlated with the starvation period and number of dead flies (Figure 2(d)). It was also observed that DG levels continued to increase during the incubation of dead male flies from 48 to 57 h, suggesting that incubation of fly corpses also causes an increase in DG levels.

### 3.3. Lipase Activity during Starvation of Male and Female Flies

**Figure 3:**
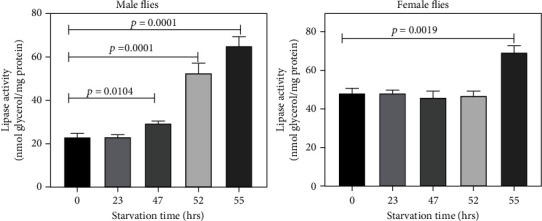
Lipase activity during starvation in 2 d old male and female flies. Lipase activity increased during the final phase of starvation in both male and female flies. Lipase activity was analyzed in triplicate. The data is presented as the mean ± SD. It was analyzed by Student's *t*-test; starvation vs. the control group.

Since we observed increased levels of DG during final phase of starvation, we also measured the activity of lipase in these flies (Figure 3). An increase in lipase activity was observed during the final phase of starvation in both male and female flies. In male flies, lipase activity increased by 27% at 47 h, 129% at 52 h, and 182% at 55 h, while in female flies, lipase activity increased by only 43% at 55 h of starvation. Similar to DG, an increase in lipase activity also continued during further incubation of dead male flies.

## 4. Discussion

To maintain metabolic homeostasis during fasting periods, metazoans have to coordinate the metabolism of glycogen, lipids, and protein, ensuring an adequate energy supply across tissues [20]. The central function of the liver in metabolic adaptation of flies is shared by the fat body, which is an important glycogen and fat storage organ in flies [21–24]. Our data shows that survival rate was different in male and female flies during starvation. Male flies died faster as compared to female flies at all three age groups studied, i.e., in 2 d, 12 d, and 23 d old flies. The female flies are larger in size than male flies, suggesting that female flies may have more nutrient storage, thus keeping them alive for a longer period upon starvation as compared to male flies. We also observed that the effect of starvation on survival of flies was age-dependent; i.e., 2 d flies survived longer than 12 d and 23 d flies. It was suggested by Aguila et al. [25] that nutrients stores acquired by the larva are transferred to the adult's larvally derived lipid stores, which may be more important to adult fitness than carbohydrate or protein stores [26]. These larval fat cells appear to be a very efficient source of nutrients as compared with the adult fat cells. This may be the reason of longer survival rate of younger flies upon starvation.

Lipids play a central role during starvation of flies. When we studied the effects of starvation on DG levels, we observed that there was an increase in the levels of DG in flies due to starvation. The increase in DG levels was much earlier in male flies as compared to female flies, which correlated with male flies dying earlier than female flies during starvation. Another interesting observation was that lipase activity also increased during starvation, which may explain the increase in DG levels in starved flies. Both DG levels and lipase activity continued to increase during further incubation when all starved flies were dead. It is possible that the increase in lipase activity during starvation may be the main cause of death in flies. Excess lipids, such as free fatty acids, are converted into neutral lipids such as TG and get stored in lipid droplets. From bacteria to humans, lipid droplets share the same blueprint: a hydrophobic core with the storage lipids is surrounded by a phospholipid monolayer with proteins attached [27]. Lipid droplets mainly serve the regulated deposition and remobilization of energy-rich neutral lipids when there is nutrients' excess and energy demand, respectively. Therefore, TG is the main source of energy in *Drosophila melanogaster* during starvation. We reported earlier that TG levels are higher in female flies as compared to male flies [19]. We measured the levels of TG in male and female flies at different ages during starvation. The levels of TG constantly decreased in both male and female flies depending upon duration of starvation. Adipokinetic- (AKH-) mediated lipolysis and brummer lipase-dependent lipolysis are involved in the regulation of TG mobilization in insects [28, 29]. AKH stimulates lipolysis of TG into DG [30]. It may be possible that in the final hours of starvation, AKH is stimulated and DG production is increased. Our data shows that lipase activity increased at the final hours in dying flies. Therefore, increased levels of DG in final hours of starvation leading to death may be due to increased activity of lipase. It seems that normal metabolism of TG constantly provided energy to flies during starvation. The increase in DG levels and lipase activity after the final phase of starvation also continued during the additional incubation period of dead male flies. In male flies, the increase in DG levels and lipase activity started at 24 h of incubation period and continued till 57 h. Therefore, our data suggests that kick off of lipase activity during starvation of flies might have started in the final phase of starvation, which continued in dead male flies during incubation; i.e., activated lipase remained active even after death of male flies, i.e., 48 h to 57 h.

In this study, we measured only neutral lipids during starvation, but other storage metabolites, such as glycogen and trehalose, may also play an important role during starvation. Adaptation to changes in food availability is a central challenge for survival. Both vertebrates and invertebrates have developed diverse physiological strategies to address both acute and chronic nutrient shortages. Starvation also induces the complete breakdown of glycogen in the fat bodies. In addition to TG, stored sugars are also utilized as energy source for survival during fasting [31]. Glycogen is the main source of glucose for energy production. In insects, glucose is stored in two different forms as the disaccharide trehalose and the branched polymer glycogen. Glycogen is synthesized and stored in several tissues, including in muscle and the fat body. Yamada et al. [31] reported that glycogen metabolism is regulated in a tissue-specific manner under starvation conditions in the fruit fly. However, the mobilization of fat body glycogen in larvae is independent of adipokinetic hormone (Akh, the glucagon homolog) but is regulated by sugar availability in a tissue-autonomous manner. Fat body glycogen plays a crucial role in the maintenance of circulating sugars, including trehalose, under fasting conditions [31].

## 5. Conclusions

Our data suggests that the increase in DG levels and lipase activity during the final phase of starvation correlates with male flies dying earlier than female flies when subjected to starvation. Therefore, the increase of lipase activity may be a possible cause of death during starvation in flies.

## Data Availability

All the data is present in the manuscript.
